# Association between Endometrial Polyps and Chronic Endometritis: Is It Time for a Paradigm Shift in the Pathophysiology of Endometrial Polyps in Pre-Menopausal Women? Results of a Systematic Review and Meta-Analysis

**DOI:** 10.3390/diagnostics11122182

**Published:** 2021-11-24

**Authors:** Amerigo Vitagliano, Mariangela Cialdella, Rossana Cicinelli, Carla Mariaflavia Santarsiero, Pantaleo Greco, Giovanni Buzzaccarini, Marco Noventa, Ettore Cicinelli

**Affiliations:** 1 Department of Women and Children’s Health, University of Padua, 35128 Padua, Italy; giovanni.buzzaccarini@gmail.com (G.B.); marco.noventa@gmail.com (M.N.); 2 Unit of Obstetrics and Gynecology, Department of Biomedical and Human Oncologic Science, University of Bari, 70124 Bari, Italy; maria.cialdella@gmail.com (M.C.); cicinelli.rossana@gmail.com (R.C.); carla.santarsiero@gmail.com (C.M.S.); ettorecicinelli@yahoo.it (E.C.); 3 Department of Translational and for Romagna Medicine, University of Ferrara, 44121 Ferrara, Italy; grcptl@unife.it

**Keywords:** chronic endometritis, endometrial inflammation, endometrial plasma cells, pre-menopausal women, endometrial polyps, polypoid endometrium, infertility, abnormal uterine bleeding, hysteroscopy, CD-138 immunohystochemistry

## Abstract

Background: Chronic endometritis (CE) and endometrial polyps (EPs) are common conditions in reproductive age women. CE is an infectious disorder of the endometrium characterized by signs of chronic inflammation at hysteroscopic and histological analyses. EPs are abnormal endometrial growths containing glands, stroma and blood vessels projecting from the lining of the uterus. During the last years, different authors have investigated the correlation between CE and EPs, with controversial results. The aim of this study was to summarize available evidence on the potential correlation between CE and EPs. Design: Systematic literature review and meta-analysis. Methods: Observational-studies were identified by searching electronic databases from their inception to September 2021. Only studies on pre-menopausal women were included. Statistical analysis was performed using MedCalc 16.4.3 (Ostend, Belgium) and Review Manager version 5.3 (Nordic Cochrane Centre, Cochrane Collaboration). The summary measures were reported as pooled proportion or odds ratio (OR) with 95% confidence interval (CI). The primary outcome was to evaluate the prevalence of CE in women with EPs. The secondary outcome was to determine the prevalence of CD-138-positive EPs among EPs. Tertiary outcomes were to compare the prevalence of CE in women with EPs versus women with a non-polypoid endometrium and to compare the prevalence of CE in women with a single EP versus women with multiple EPs. Results: Eight observational studies (n = 3225 patients) were included in quantitative synthesis. Pooled prevalence of CE among women with EPs was 51.35% (95% CI, 27.24–75.13%). Pooled proportion of CD-138-positive EPs among EPs was 70.73% (95% CI, 55.73–83.68%). Women with EPs showed higher prevalence of CE compared to women without EPs (OR 3.07, 95% CI 1.59–5.95). Women with ≥3 EPs had higher prevalence of CE then women with a single EP (OR 3.43, 95% CI 1.83–6.46). Conclusions: In pre-menopausal women, CE and EPs may have a dependent relationship and may represent two consequent steps of a common pathological process.

## 1. Introduction

Chronic endometritis (CE) is a chronic inflammatory condition of the endometrium, whose diagnosis is currently based on the demonstration of plasma cell infiltration within endometrial stromal tissue [1,2,3,4,5,6].

The pathogenesis of CE may be the result of qualitative and quantitative alteration of endometrial microbiome. According to recent studies, the main species of bacteria involved in the development of CE are Enterococci, Streptococci, Staphylococci, Mycoplasma spp, Gardnerella vaginalis, Ureaplasma urealyticum, Chlamydia trachomatis and Neisseria gonorrhoeae [7,8,9,10,11,12].

Clinically, CE is a subtle pathology accompanied by only mild and unspecific disturbances, such as abnormal uterine bleeding (AUB), dyspareunia, pelvic discomfort and leukorrhea. Nevertheless, this condition may impair endometrial receptivity, resulting in unexplained infertility, recurrent spontaneous abortion (RSA) and repeated implantation failure (RIF) at IVF [5,13,14,15,16,17,18,19,20,21].

In 2005, Cicinelli et al. first demonstrated that CE was associated with small mucosal proliferations (<1 mm in diameter) which were similar to the endometrial polyps (EPs) at hysteroscopy, defined by the authors as “micropolyps” [22,23]. This finding was subsequently confirmed by several studies [7,24,25,26].

EPs are localized, sessile or pedunculated projections of endometrial mucosa, resulting from the hyperplastic overgrowths of glands and stroma around a vascular core [27,28]. EPs are common findings in both reproductive age and post-menopausal women. While the development of micropolyps has been definitely ascribed to chronic endometrial inflammation, the correlation between EPs (i.e., “macropolyps”) and CE remains unclear. Although some studies have suggested a potential link between CE and EPs [29], currently, the pathogenesis of EPs is still believed to be mainly related to hormonal factors [30]. As the relationship between CE and EPs is far from being clear, the aim of this review was to summarize the available evidence on the correlation between CE and EPs.

## 2. Materials and Methods

### 2.1. Study Design

This is a systematic review of published data. The review was reported following the Preferred Reporting Items for Systematic Reviews and Meta-Analyses (PRISMA) guidelines [31].

As it was a review of published data, institutional review board approval was not required.

### 2.2. Search Strategy

Electronic databases (ScienceDirect, Medline, Scopus, Embase, the Cochrane library, Clinicaltrials.gov, EU Clinical Trials Register and the World Health Organization International Clinical Trials Registry) were searched until 30 September 2021 (without date restriction).

Key search terms were chronic endometritis OR endometrial inflammation OR endometrial plasma cells AND endometrial polyps OR endometrial proliferation OR endometrial lesions OR polypoid endometrium OR endometrial polyposis. The electronic search and the eligibility of the studies were independently assessed by two of the authors (A.V., R.C.).

### 2.3. Inclusion Criteria

All the studies assessing the association between CE and EPs were evaluated. All studies (experimental and observational) reported in the English language were eligible. Meeting abstracts were also eligible for inclusion. CE was defined according to the criteria applied in the original articles. Studies evaluating other types of endometrial inflammation (such as acute, subacute or tubercular endometritis) were excluded.

### 2.4. Study Selection and Data Extraction

Two authors (A.V., M.C.) independently assessed the inclusion criteria and study selection. Disagreements were discussed with a third reviewer (E.C.).

Data extraction was performed by two independent investigators (R.C., C.M.S.). Data extracted included study features (design, setting, objectives, main findings), population characteristics (age, inclusion criteria, pre-menopausal/post-menopausal, infertile/fertile) and the criteria for CE diagnosis. When studies involved a control group considered negligible for the endpoints of the review, inherent data was not extracted. A manual search of reference lists of studies was performed to avoid missing relevant publications. One author (E.C.) reviewed the selection and data extraction process. The results were then compared, and any disagreement discussed and resolved by consensus.

### 2.5. Study Outcomes

The primary outcome of this study was to evaluate the prevalence of CE in women with EPs. The secondary outcome was to determine the prevalence of CD-138-positive EPs among EPs.

Tertiary outcomes were to compare the prevalence of CE in women with EPs versus women with a non-polypoid endometrium and to compare the prevalence of CE in women with a single EP versus women with multiple EPs.

### 2.6. Data Synthesis and Analysis

We reported all descriptive characteristics of studies, including study design, study aim, year of publication, study setting, type and number of patients, criteria and techniques for achieving CE diagnosis, and study results. Data analysis was completed by two authors (A.V., M.N.). For the primary and secondary outcomes, statistical analysis was performed using MedCalc 16.4.3 (Ostend, Belgium). The proportion method was used, and a forest plot was derived for meta-analysis. The proportion of patients was analyzed at a 95% confidence interval (CI). For the tertiary outcomes, the analysis was conducted using Review Manager version 5.3 (Nordic Cochrane Centre, Cochrane Collaboration. The study outcomes were expressed using odds ratios (ORs) with 95% confidence intervals (95% CIs). *p* values lower than 0.05 were statistically significant. The I^2^ statistics were used to assess heterogeneity. Degree of heterogeneity was considered as low when I^2^ was <30%, moderate if between 30% and 50%, and high if I^2^ was >50%. A random-effects model (DerSimonian and Laird method) was applied to meta-analyses. Subgroup and sensitivity analysis were also planned in order to explore the sources of heterogeneity across studies (when at least three studies when included in meta-analysis).

## 3. Results

### 3.1. Study Selection

The literature search initially identified 1264 articles after removing duplicates. The titles of these manuscripts were screened, resulting in 75 studies potentially eligible for inclusion. Of the manuscripts identified, 61 studies were excluded after the evaluation of the abstracts, and 14 studies were further evaluated. Six studies were excluded after the evaluation of full text (see Table A1 and Table A2) [4,9,30,32,33,34].

**Figure 1:**
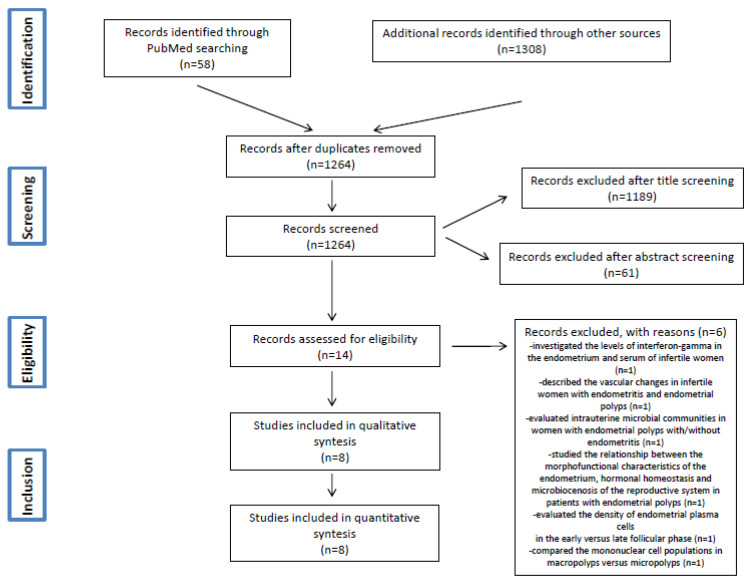
PRISMA Flow Diagram.

Finally, a total of eight studies were included in this present review [29,35,36,37,38,39,40,41]. The study flow diagram is displayed in Figure 1.

### 3.2. Included Studies

All the studies were monocentric. Five were retrospective cohort or case-control studies. Two were cross-sectional studies. Sklyarova et al. 2020 did not report any information about the design of their study [35]. Main characteristics of included studies, patients and study aims are summarized in Table 1 and Table 2.

#### 3.2.1. Patients

The total number of patients evaluated was 3225. Studies included patients with reproductive issues (infertility, recurrent miscarriage), AUB or miscellaneous populations undergoing hysteroscopic procedures.

#### 3.2.2. Diagnosis of CE

In all studies, the diagnosis of CE was based on the demonstration of endometrial plasma cells using CD-138 immunohistochemistry. Diagnostic criteria for CE varied among studies. The most common criteria applied were the detection of ≥5 plasma cells in 10 HPF [29,35,36,39] and ≥1 plasma cells in 10 HPF [37,40,41] using CD-138 immunostaining (See Table 1).

### 3.3. Synthesis of Results

#### 3.3.1. Primary Outcome: Prevalence of CE in Women with Eps

**Figure 2:**
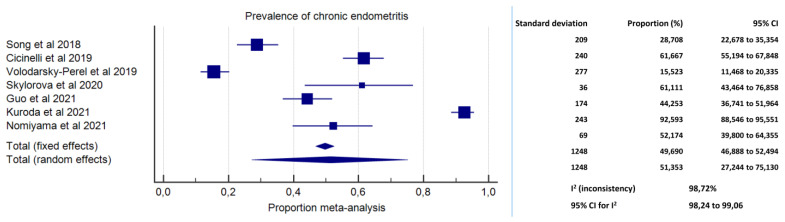
Forest plot. Prevalence of chronic endometritis in pre-menopausal women with endometrial polyps.

Meta-analysis on the prevalence of CE in women with EPs was conducted based on data from seven studies [29,35,37,38,39,40,41]. A total number of 1248 patients with EPs were included. The prevalence of CE ranged from 28.71% in the study by Song et al. [37] to 92.59% in the study by Kuroda et al. [29]. The pooled proportion was 51.35% (95% CI, 27.24%–75.13%; I^2^ = 98.72%) (Figure 2).

#### 3.3.2. Secondary Outcome: Prevalence of CD-138-Positive EPs among EPs

**Figure 3:**
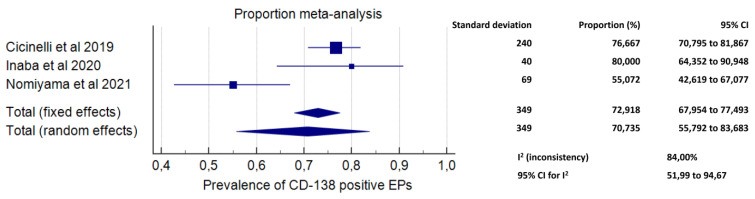
Forest plot. Proportion of CD-138 immunoreactive endometrial polyps.

Meta-analysis included data from three studies. A total number of 349 EPs were analysed [36,38,40]. The prevalence of CD-138-positive EPs among EPs ranged from 55.07% in the study by Nomiyama et al. [38] to 80.00% in the study by Inaba et al. [36]. The pooled proportion was 70.73% (95% CI, 55.73–83.68%; I^2^ = 84.00%) (Figure 3).

#### 3.3.3. Tertiary Outcomes

Prevalence of CE in women with EPs compared to women with a non-polypoid endometrium

**Figure 4:**
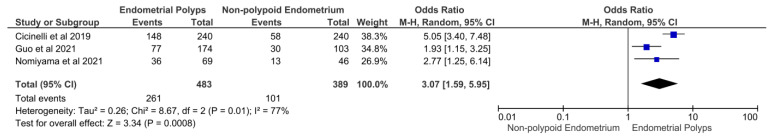
Forest plot. Women with endometrial polyps versus women with a non-polypoid endometrium: prevalence of chronic endometritis.

Data on 872 women (n = 483 with EPs and n = 389 with a non-polypoid endometrium) from three studies [38,39,40] showed a significantly higher prevalence of CE in women with EPs as compared to women with a non-polypoid endometrium (OR 3.07, 95% CI 1.59–5.95, I^2^ = 77%, *p* = 0.0008; Figure 4).

#### 3.3.4. Prevalence of CE in Women with a Single EP Versus Women with Multiple EPs

Meta-analysis was not feasible for this outcome. Data on 174 women (n = 92 with multiple EPs [≥3]; n = 82 with a single EP) from a single study [39] revealed a significantly higher prevalence of CE in women with multiple EPs compared to women with a single EP (OR 3.43, 95% CI 1.83–6.46, *p* = 0.0001; data not shown).

#### 3.3.5. Investigation of Sources of Heterogeneity across Studies

Sub-analyses were feasible for the primary outcome of our study. Splitting the analyses based on the diagnostic criteria for CE did not reduce the statistical heterogeneity. Meta-analysis of studies in which CE was diagnosed with ≥5 plasma cells in 10 HPF (n = 486 patients) [29,35,36,39] found a pooled prevalence of CE of 65.54% (95% CI 27.03%–94.77%; I^2^ = 98.62%; data not shown). Differently, meta-analysis of studies in which the diagnosis of CE was based on the identification of ≥1 plasma cells in 20 HPF (n = 706 patients) [37,40,41] found a pooled prevalence of CE of 34.25% (95% CI 10.60–63.18%; I^2^ = 98.46%; data not shown). Secondary analyses based on population characteristics were not feasible.

## 4. Discussion

Although the knowledge about endometrial physiology and pathology has improved over the last decade, the pathogenesis of EPs remains partly unexplained [33,39,42]. Likewise, the relevance of CE as a chronic inflammatory disorder of the endometrium, as well as its etiopathology are still uncertain [43,44]. What appears clearer is that both EPs and CE are common pathologies in pre-menopausal women suffering from reproductive disorders [29,35,38]. This observation has given rise to the suspicion that EPs and CE may represent causative factors of a defective endometrial receptivity [11,14,16,17,45,46]. Additionally, a potential pathogenetic link between EPs and CE was recently postulated [33,38,40].

Chronic inflammation has been identified as a causative factor in the development of polyps in different mucosal tissues across the human body (i.e., urinary tract, upper respiratory tract and lower gastrointestinal tract) [47,48,49]. With regard to EPs, a potential inflammation-driven pathogenesis has been long neglected by the scientific community. Here, we have provided a first summary of evidence on the possible correlations between CE and EPs in pre-menopausal women.

### 4.1. Main Results and Implications

We found high prevalence of CE in pre-menopausal women with EPs (51.35%; 95% CI, 27.24%–75.13%), with high inconsistency (I^2^ = 98.72%). Arguably, the high variability in CE prevalence across studies was ascribable to heterogeneity in the diagnostic criteria for CE and in populations’ characteristics. High inconsistency, however, limits the overall accuracy of the prevalence estimates. Yet, we believe the data present a compelling argument for a renewed appraisal, with at least one out of four pre-menopausal women with EPs revealed to have concomitant CE (up to three women based on the 95% CI) (Figure 2).

The results of the primary outcome were supported by secondary findings of our study (Figure 3 and Figure 4). Indeed, exposure to EPs was associated with higher odds of being affected by CE compared to a non-polypoid endometrium (OR 3.07, 95% CI 1.59–5.95; *p* = 0.0008), and exposure to multiple EPs increased the odds of CE compared to a single EP (OR 3.43, 95% CI 1.83–6.46, *p* = 0.0001). Additionally, an extremely high prevalence of CD-138 immunoreactive plasma cells (i.e., the typical markers of CE) within the EP tissue was found (70.73%; 95% CI, 55.73–83.68%).

The findings from this present meta-analysis are in agreement with the results of our previous study showing an association between CE and EPs [40]. The pooled percentage of CD-138-positive EPs were similar (i.e., 70.73% and 76.7% in this review and in our previous study [40], respectively). Notably, in our previous study [40], we also found that women with CD-138-positive EPs were more likely to suffer from CE compared to those with CD-138 negative EPs (64.1% vs. 30.7%; *p* < 0.0001). The study by Kitaya et al. [4] was in partial contrast with these data. In their prospective cross-sectional study on infertile patients undergoing hysteroscopy, the authors reported no plasma cell infiltration within specimens from EPs. Additionally, plasma cell density was significantly higher in micropolypoid tissue compared to EP tissue. Nevertheless, as per authors’ admission, the sample size of the study was small (n = 23 women with EPs). Moreover, that study had no epidemiological purposes, but aimed exclusively to compare the mononuclear cell infiltration in macropolyps compared to micropolyps. Thus, women with EPs and concomitant signs of CE at hysteroscopy (e.g., micropolyps) were plausibly excluded from the study.

If we depict a connecting line between the results here presented, an interdependent correlation between EPs and CE may be postulated. In particular, EPs and CE could represent two consequent steps of a common pathological process. Such a theory was first proposed by Carvalho et al., in 2013 [33]. The authors conducted a descriptive histological study on endometrial samples from 435 infertile women. Among those patients, the 24.6% were diagnosed CE, and the 15.9% showed histological changes that were highly suspicious for CE. Interestingly, the authors observed specific endometrial vascular changes that were common in CE and EPs. In particular, 70% of vascular alterations in CE corresponded to vessel wall hyaline thickening, with similar morphology to the thick-walled vessels along the vascular axis of EPs. Moreover, the alteration was frequently associated with thrombi and/or fibrinoid degeneration of the vessel wall, potentially suggesting a vasculopathy caused by inflammation.

From a molecular point of view, chronic inflammation may promote EPs development by distorting the signaling pathways that control endometrial tissue proliferation. In this respect, we recently found an altered endometrial expression of genes involved in inflammatory, cell proliferation, and apoptosis processes in women with CE (including vascular endothelial growth factors [A, B, C], epidermal growth factor, tumor necrosis factor, interferon-ℽ, transforming growth factor β-1, cell division control protein variant, cyclin D3, cyclin B1, BCL-2-associated X protein, BCL-2 associated X protein transcript variant alpha and interleukin-12), with a dominance of proliferative and anti-apoptotic activity [50]. These factors in CE may potentially justify the gradual development of endometrial proliferative lesions emerging from a scenario of chronic inflammation. Notably, similar pathogenic pathways have been previously highlighted with regard to the development of colorectal polyps in patients with inflammatory bowel diseases [51].

A pathogenetic link between CE and EPs is supported by indirect observations, too. In a retrospective study on 323 infertile women, Sun et al. [52] found a significantly higher prevalence of EPs in women with fallopian tube obstruction compared to those with patent fallopian tubes (42.9% vs. 20.1%, *p* < 0.0001), suggesting a correlation between EPs and history of pelvic inflammatory disease (PID). Likewise, in women with hydrosalpinx, which is a frequent complication of PID, an increased risk of CE has been demonstrated [53]. Common actors in the development of all these conditions may be some infectious agents. Notably, quantitative and qualitative alterations of endometrial microbiome were clearly demonstrated in CE, with a dominance of gram negative and intracellular bacteria [54,55,56]. In women with EPs, significant alterations of endometrial microbiome were also found (i.e., with increased percentages of Lactobacillus, Bifidobacterium, Gardnerella, Streptococcus, and Alteromonas and decreased expression of Pseudomonas) [9]. However, those changes in endometrial microbiome were not definitely attributed to causative bacteria of CE.

Last but not least, CE and EPs commonly coexist with endometriosis. Endometriosis is a chronic, estrogen-dependent disorder characterized by inflammatory reaction in the ectopic and eutopic endometrium [57,58,59]. Endometriosis is a major cause of infertility and recurrent pregnancy loss after natural and medically assisted conception [60,61]. In a previous study, we found high prevalence of CE in severe endometriosis compared to controls (38.5% vs. 14.1%) [62]. Our findings were in line with those from other studies on different ethnic groups [63,64]. Interestingly, infertile women with endometriosis also show higher prevalence of EPs compared to controls without endometriosis (68.35% vs. 20.51%; Shen et al. study [64]). Theoretically, the correlation between endometriosis, CE and EPs may rely on common infectious and inflammatory factors [65]. In women with endometriosis, Khan et al. [66] found high levels of lipopolysaccharides (LPS) in peritoneal fluids and high contamination of Esherichia coli and LPS in the menstrual effluents. The “hypothesis of bacterial contamination in endometriosis” implies an initial bacterial stimulus, likely with Gram-bacteria (due to their high LPS content), followed by sustained inflammation, which allows establishing a vicious circle involved in the development of endometriosis [65,66,67]. In line with this theory, Chadchan et al. [68] recently found that the administration of broad-spectrum antibiotics led to size reduction of endometriotic lesions in murine models. However, even if a common thread between endometriosis, CE, and EPs is possible, we must recognize that current evidence is insufficient to draw firm conclusions.

Our findings may have several future clinical and research implications. In pre-menopausal women with EPs undergoing hysteroscopy, physicians may be advised about the need of excluding CE diagnosis through a careful inspection of the uterine cavity and by undertaking targeted endometrial samples of the non-polypoid endometrium. This suggestion may be especially relevant in those patients with infertility or recurrent pregnancy loss, in whom CE persistence after surgery may be associated with poor reproductive prognosis. The investigation of CE may also be relevant for those women who are not seeking pregnancy, as the persistence of CE after polypectomy may theoretically increase the risk of EP recurrence in the long term. When CE is concomitant with EPs, the administration of antibiotics may be considered in addition to polypectomy. In this respect, a recent study by Kuroda et al. [29] concluded that empiric antibiotic therapy with doxicicline (200 mg daily for 14 days) in addition to polypectomy should be discouraged as it reduced the recovery rate from CE as well as the clinical pregnancy rate within six months in a group of infertile women undergoing IVF [29]. However, these results are not conclusive for many reasons, including the observational study design, the short-term follow-up, and the lack of data about live births. Additionally, a “scratching effect” due to hysteroscopy immediately prior to IVF [69,70,71] could not be excluded. Thus, the administration of antibiotic therapy in addition to polypectomy in infertile women with concomitant CE prior to infertility treatments needs future investigation. Similarly, the role of antibiotic therapy against CE for the prevention of EPs recurrence after surgery is still undefined.

### 4.2. Strengths and Limitations

To the best of our knowledge, this is the first review investigating the association between EPs and CE in pre-menopausal women. Rigorous methodology and the inclusion of quantitative data synthesis are additional points of strength of this study. The main limitations of this study are inherent to the limitations of included studies, including some disparities in the diagnostic criteria for CE, population characteristics and study design.

## 5. Conclusions

This review found high prevalence of CE in pre-menopausal women suffering from EPs. Moreover, the majority of EPs showed plasma cell infiltration at CD-138 immunohistochemistry.

The risk of CE was higher in women with EPs compared to women with a non-polypoid endometrium, as well as in those with three or more EPs compared to those with a single EP.

Based on available evidence in pre-menopausal women, CE and EPs may have a dependent relationship and may represent two consequent steps of a common pathological process. However, a conclusive pathogenetic association between these two entities has not been demonstrated yet.

Future studies investigating the occurrence of EPs in pre-menopausal women with a prior diagnosis of CE are necessary. Moreover, in women undergoing hysteroscopic polypectomy, future randomized controlled trials will assess the role of antibiotic therapy against CE for improving the success of IVF cycles, as well as for preventing the recurrence of EPs in the long term.

## Figures and Tables

**Table 1:** General features of included studies.

Study ID	Study Design	Country	Patients (Number)	Women Characteristics	CE Definition	Study Outcomes
Song et al. 2018 [37]	Retrospective cohort study	China	1551	Premenopausal women with abnormal uterine bleeding or reproductive failure.	≥1 CD-138 positive plasma cell per 10 HPF	To examine the prevalence of chronic endometritis in a consecutive series of endometrial biopsies and to identify confounding variables that may affect the prevalence of chronic endometritis
Cicinelli et al. 2019 [40]	Retrospective case-control study	Italy	480	Premenopausal women with AUB. **Group A:** n = 240 women with EPs (diagnosed at hysteroscopy and histology) **Group B:** included 240 patients without evidence of EPs at hysteroscopy.	>1 CD-138 positive plasma cell per 10 HPF	To investigate the correlation between endometrial polyps (EPs) and chronic endometritis (CE)
Volodarsky-Perel et al. 2019 [41]	Retrospective cohort	Canada	277	Patients undergoing hysteroscopic polipectomy **Group A:** Infertile (n = 137) **Group B:** Fertile (n = 140)	≥1 plasma cells per 10 HPF	(1) To evaluate the prevalence of CE in infertile women with EPs compared with infertile women with EPs (2) To investigate the prevalence of CE in women with primary infertility compared with those with secondary infertility
Inaba et al. 2020 [36]	Retrospective case-control study	Japan	40	4 groups of 10 patients each by the shape of the polyp (sessile type or pedunculated type) and Dienogest treatment prior to the operation	>5 CD138-positive cells per 10 HPF	To investigate the effects of Dienogest on the proliferation and inflammation of endometrial polyps
Sklyarova et al. 2020 [35]	na	Ukraine	133	Reproductive age women with reproductive health disorders **Group I:** 30 patients with recurrent pregnancy loss **Group II:** 47 women with primary infertility **Group III:** 36 women who had a polyp or endometrial polyps detected during routine ultrasound. **Control group:** 20 women	na	To analyze the incidence of chronic endometritis in women of reproductive age with reproductive health disorders
Guo et al. 2021 [39]	Cross-sectional study	China	277	Premenopausal patients who have undergone hysteroscopic inspection with gynecologic conditions for different reasons **Group A: single EP:** n = 82 **Group B: ≥6 EPs:** n = 92 **Control group:** n = 103	≥5 CD138-positive cells in 10 HPF	To determine whether single endometrial polyp (EP) or multiple EPs (polyp number ≥ 6) are associated with CE
Kuroda et al. 2021 [29]	Cross-sectional study	Japan	222	Infertile patients undergoing hysteroscopic polipectomy -**Group A:** women with CE who received doxicicline after polypectomy: n = 62 -**Group B:** women with CE who did not receive doxicicline after polypectomy: n = 160.	≥5 CD138-positive cells in 10 HPF	To compare the therapeutic effects of hysteroscopic polypectomy with and without doxycycline treatment on CE
Nomiyama et al. 2021 [38]	Retrospective cohort study	Japan	245	Women with a suspicion of EPs undergone diagnostic hysteroscopy **Group 1:** 38 patients with CD138 + EPs **Group 2:** 31 patients with CD138 − EPs **Group 3:** no EPs	≥10 CD138-positive cells in 20 HPF	To determine the prevalence of CE in groups 1, 2 and 3

CE: chronic endometritis; EPs: endometrial polyps; SD: standard deviation; na: not assessed; HPF (high-power-fields); +: positive; −: negative: Study in abstract form.

**Table 2:** Main findings of included studies.

Study ID	Main Findings
Song et al. 2018 [37]	- The prevalence of CE was increased in women with recurrent implantation failure, abnormal uterine bleeding, and endometrial hyperplasia compared with those without the respective conditions, and also significantly higher in the proliferative stage of the menstrual cycle compared with the luteal phase. Women with EPs had a global prevalence of CE of 28.7%. ©
Cicinelli et al. 2019 [40]	- EPs were commonly associated with CE in the premenopausal women suffering from AUB (64.1%). - Moreover, the majority of EPs were positive for CD-138 staining (76.7%), suggesting a possible hidden association between chronic inflammation and EPs. ©
Volodarsky-Perel et al. 2019 [41]	- The prevalence of CE in the group of infertile women was significantly higher than that in the control group (22.6% vs. 8.6%; *p* = 001). - Women with primary infertility and those with secondary infertility showed no difference in CE prevalence. ©
Inaba et al. 2020 [36]	- Dienogest prescription prior to hysteroscopic surgery of EPs has inhibitory effects on cellular proliferation. - Patients with EPs might be significantly complicated by CE. Eighty percent of EPs showed CD-138 positivity. ©
Sklyarova et al. 2020 [35]	In patients with habitual miscarriage, primary infertility and women in the planning of pregnancy and endometrial polyps, a high frequency of bacterial vaginosis, and recurrent inflammatory diseases of the lower parts of the reproductive system chronic endometritis were noted (*p* > 0.01). In immunohistochemical examination of the endometrium, CE was diagnosed in 80% of patients with habitual miscarriage, in 55% of women with primary infertility and in 61% of women when planning pregnancy and EPs (*p* > 0.01). ©
Guo et al. 2021 [39]	Multiple EPs were positively associated with CE among reproductive-aged women (58.7%) compared to single EP (28%) and controls (29.1%), suggesting a possible hidden etiopathogenetic link between chronic inflammation and multiple EPs. ©
Kuroda et al. 2021 [29]	CE was present in 92.6% of women with EPs. Most CE patients with endometrial polyps had been cured by polypectomy without doxycycline (88.8% vs. 58.1%). Clinical pregnancy rate within 6 months was higher in women who did not receive antibiotics (63.2% vs. 43.8%). ©
Nomiyama et al. 2021 [38]	Infertile patients with EPs have higher prevalence of CE compared to those without EPs. Women with CD-138-positive EPs have higher rate of CE compared to those with CD-138-negative EPs and those without EPs (68.4% vs. 32.2% vs. 28.3%). ©

© Full-text studies. CE: chronic endometritis EPs: endometrial polyps; AUB: abnormal uterine bleeding.

## Appendix A

**Table A1:** General features of the studies excluded after full text screening.

Study ID	Study Design	Country	Patients (Number)	Women Characteristics	CE Definition	Study Outcomes
Mollo et al. 2011 [32]	Prospective controlled	Italy	42	-Infertile women Study group: n = 21 women affected by EPs Control group: n = 21 women without EPs	na	To assess levels of interferon-gamma (IFN-g) both in serum and in endometrial biopsy samples from infertile patients with EPs as compared to women without EPs
Kitaya et al. 2012 [4]	Cross-sectional	Japan	75	-Infertile women with 3 or more IVF failures Macropolypoid endometrium group: n = 23 Micropolypoid endometrium group: n = 25 Nonpolypoid endometrium group: n = 27	≥5 stromal CD138 + plasmacytes in 20 HPF	To characterize the local mononuclear cell subsets in infertile patients with endometrial macropolyps versus micropolyps
Carvalho et al. 2013 [33]	Prospective cohort	Brazil	435	Infertile patients	High vascular density with endothelial proliferation and swelling, hyaline thickening of the vessel wall with luminal occlusion, fibrinoid degeneration of the vessel wall and small vessel thrombosis	To describe morphological vascular changes in endometrial samples from asymptomatic infertile patients and their association with CE and EPs
Fang et al. 2016 [9]	Prospective controlled	China	30	Infertile women Group A (EPs)+: n = 10 Group B (EPs+CE)+: n = 10 Control group: n = 10	≥5 CD138-positive cells in 10 HPF	To characterize the intrauterine microbial communities in patients suffering from endometrial polyps combined with or without chronic endometritis and the intrauterine population difference compared to healthy donors
Kosei et al. 2017 [30]	na	Georgia	130	Group A (EPs): n = 34 Group B (micropolyps): n = 30 Group C (EPs + micropolyps): n = 36 Control Group: n = 30 healthy women	na	To study the relationship between the morphofunctional characteristics of the endometrium, hormonal homeostasis and microbiocenosis of the reproductive system in patients with endometrial polyps
Tolani et al. 2020 [34]	Retrospective cohort study	USA	201	Women with infertility or recurrent miscarriage Group A: Normal uterine cavity n = 167 Group B: Abnormal uterine cavity n = 34	≥1 plasma cells per 10 HPF	To compare plasma cell infiltrate in patients with normal and abnormal cavity evaluations

CE: chronic endometritis; EPs: endometrial polyps; na: not assessed; HPF (high-power fields); +: positive.

**Table A2:** Main findings of the studies excluded after full text screening.

Study ID	Main Findings
Mollo et al. 2011 [32]	Higher concentrations of IFN-ℽ were detected in the serum and the endometrium of infertile patients with EPs. The possible role of an inflammatory factor in a proliferative pathology represents a novel insight into the understanding of EPs and their relationship with infertility.
Kitaya et al. 2012 [4]	Compared with the non-polypoid endometrium, macropolypoid endometrium contained a lower density of pan-leukocytes, pan-T cells, and NK cells, whereas micropolypoid endometrium had a higher density of pan-leukocytes and B cells, along with a lower density of NK cells.
Carvalho et al. 2013 [33]	Endometrial samples from infertile patients present a broad spectrum of vascular changes, most of them associated with CE. This association is also identified in EPs. It is possible that the vessel axis of functional polyps may originate from the evolution of the vascular changes associated with CE. This would place EPs among the spectrum of inflammatory endometrial diseases.
Fang et al. 2016 [9]	Uterine microbiomes between patients with EP and the healthy are significantly different, and all the potentially important variations of uterine microbes may cause EP, but not definitively related to CE.
Kosei et al. 2017 [30]	Progesterone deficiency and local immune imbalance with severe hypofunctional NK cells against viral and fungal infestations result in excessive endometrial cell proliferation and development of an isolated polyp.
Tolani et al. 2020 [34]	Intracavitary pathology is associated with a significantly higher frequency and density of endometrial plasma cells (71% vs. 41%).

CE: chronic endometritis; EPs: endometrial polyps; IFN: interferon; NK: natural killer.
